# Muscle and Hip Contact Forces in Asymptomatic Men With Cam Morphology During Deep Squat

**DOI:** 10.3389/fspor.2021.716626

**Published:** 2021-09-09

**Authors:** Danilo S. Catelli, Erik Kowalski, Paul E. Beaulé, Mario Lamontagne

**Affiliations:** ^1^Human Movement Biomechanics Laboratory, School of Human Kinetics, Faculty of Health Sciences, University of Ottawa, Ottawa, ON, Canada; ^2^Division of Orthopaedic Surgery, Faculty of Medicine, University of Ottawa, Ottawa, ON, Canada

**Keywords:** femoroacetabular impingement, cam morphology, biomechanics, squat, muscle forces, hip contact forces

## Abstract

Cam morphology is defined as an aspherical femoral head-neck junction that causes abnormal contact of the acetabular rim with the anterior hip. Imaging confirmation of the cam morphology, associated with clinical signs and pain in the hip or groin, is characterized as femoroacetabular impingement (FAI) syndrome. Although some individuals with cam morphology do not experience any symptoms, sparse studies have been done on these individuals. Understanding the way asymptomatic individuals generate muscle forces may help us to better explain the progression of the degenerative FAI process and discover better ways in preventing the onset or worsening of symptoms. The purpose of this study was to compare the muscle and hip contact forces of asymptomatic cam morphology (ACM) and FAI syndrome men compared to cam-free healthy controls during a deep squat task. This prospective study compared 39 participants, with 13 in each group (ACM, FAI, and control). Five deep squatting trials were performed at a self-selected pace while joint trajectories and ground reaction forces were recorded. A generic model was scaled for each participant, and inverse kinematics and inverse dynamics calculated joint angles and moments, respectively. Muscle and hip contact forces were estimated using static optimization. All variables were time normalized in percentage by the total squat cycle and both muscle forces and hip contact forces were normalized by body weight. Statistical non-parametric mapping analyses were used to compare the groups. The ACM group showed increased pelvic tilt and hip flexion angles compared to the FAI group during the descent and ascent phases of the squat cycle. Muscle forces were greater in the ACM and control groups, compared to the FAI group for the psoas and semimembranosus muscles. Biceps femoris muscle force was lower in the ACM group compared to the FAI group. The FAI group had lower posterior hip contact force compared to both the control and ACM groups. Muscle contraction strategy was different in the FAI group compared to the ACM and control groups, which caused different muscle force applications during hip extension. These results rebut the concept that mobility restrictions are solely caused by the presence of the cam morphology and propose evidence that symptoms and muscle contraction strategy can be the origin of the mobility restriction in male patients with FAI.

## Introduction

Femoroacetabular impingement (FAI) syndrome has become a common cause for athletic hip injuries, which result in chondrolabral damage and early hip joint degeneration (Ganz et al., 2003, 2008; Agricola et al., 2013). The cam-type hip morphology is defined by an aspherical femoral head-neck that produces elevated alpha angles and decreased anterior head-neck offsets (Nötzli et al., 2002), and can result in anterior hip or groin pain, labral tears, and damage to the acetabular articular cartilage (Ito et al., 2001; Gosvig et al., 2008; Bowman et al., 2010; Speirs et al., 2013). Previous studies on FAI pathomechanism have speculated the existence of the cam-type morphology as causative to limit the functional range of motion (ROM), which has been shown in patients with cam-type FAI who demonstrated less hip and pelvic ROM during gait (Kennedy et al., 2009; Rylander et al., 2011; Brisson et al., 2013; Diamond et al., 2016; Ng et al., 2018b; Catelli et al., 2019b; Savage et al., 2021), deep squat (Lamontagne et al., 2009, 2011; Ng et al., 2015; Bagwell et al., 2016; Catelli et al., 2019a, 2020), stairs (Rylander et al., 2013; Diamond et al., 2018; Catelli et al., 2019a, 2021), and clinical assessments (Kapron et al., 2012; Ng et al., 2016). Recent *in silico* analyses have demonstrated reduced hip contact forces (HCFs) compared to matched healthy individuals while performing different tasks (Catelli et al., 2019b, 2020, 2021). However, the increasing reports of individuals with cam-type morphology who do not show clinical signs or symptoms (Hack et al., 2010; Jung et al., 2011; Ng et al., 2015, 2018a; Catelli et al., 2018; Graffos et al., 2020) are an indication that the cam-type morphology alone may not fully justify symptoms of an individual or limited ROM. Understanding the way individuals with ACM move may help us better explain the causality of hip pain and progression of degenerative FAI process and discover better ways in preventing the onset or worsening of the symptoms.

The purpose of this study was to compare the muscle and hip contact forces of ACM and FAI syndrome men compared to cam-free healthy control (CTRL) individuals during a deep squat task. It is hypothesized that the ACM kinematics, kinetics, and muscle forces outputs will resemble the CTRL levels, instead of the FAI. However, based on analysis of different tasks (Ng et al., 2018b; Catelli et al., 2019b, 2020, 2021), we expect the CTRL will still produce higher HCF compared to the other groups.

## Materials and Methods

This prospective study compared three participant groups (symptomatic, asymptomatic, and healthy cam-free individuals) during cross-sectional observations of their squatting kinematics and kinetics that fed a computational modeling approach to estimate hip muscle forces and contact loading. The study protocol was approved by the University and hospital research ethics boards. Participants provided informed consent, and investigations were conducted ethically in conformity with research principles.

### Participants

A 2-year recruitment process initially selected 68 participants for this study. Twenty-two of them (two women) presented themselves to the senior orthopedic surgeon with unilateral hip pain, clinical impingement signs, and a cam-type morphology (alpha angles > 50.5° anteriorly at the 3:00 clock-face position about the femoral neck, or 60° anterosuperiorly at 1:30) (Nötzli et al., 2002; Rakhra et al., 2009; Barton et al., 2011) were classified as patients with FAI and scheduled for surgery. Forty-six participants (six women) were recruited from the community to serve as controls. Their initial radiographs were taken to screen for the presence of a cam-type morphology, which was confirmed positive for several individuals; however, they did not experience any clinical symptoms. After this finding, the decision was made to have all participants (with and without the cam-type morphology) undergo full radiographic screening using low-dose CT. Each participant underwent diagnostic CT (Aquilion, Toshiba Medical Systems, Japan or Discovery CT750, GE Healthcare, Mississauga, Canada) to confirm the presence of the cam-type morphology. Physical examinations and impingement tests (i.e., flexion-adduction and internal rotation - FADIR, flexion-abduction and external rotation - FABER) were performed to confirm the presence of symptoms. The asymptomatic individuals remained blinded and were unaware if they had a cam-type morphology until after the completion of the study. As a matter of comparison, the affected hip in patients with FAI with a bilateral cam-type morphology was the one with greater clinical signs (i.e., surgical side); in the ACM group, the side of interest was the one with the larger alpha angle, and the selected hip for the CTRL participants was based on their dominant leg (i.e., preferred leg to kick a soccer ball).

Participants were excluded from this study if they had a body mass index (BMI) higher than 30 kg/m^2^ or any other hip or spine deformity, musculoskeletal abnormality, major lower-limb and spinal injuries, or surgery. As a visual strategy to standardize the characteristics of the deep squat, participants were also excluded in case they were unable to surpass 90° of knee flexion during the task. Due to the low number of female individuals recruited, we decided to include only male participants in this study. As a result, five participants were excluded for a high BMI (two FAI, two CTRL, one ACM); one FAI for previous knee surgery; one FAI for low back pain; one FAI for CT technical issues; one FAI and one ACM for data collection complications; one FAI, two ACM, and two CTRL did not perform a minimum of 90° of knee flexion during squat; and two FAI, three ACM, and three CTRL female participants were excluded. In all, 45 participants met the inclusion criteria. To age-, BMI-match the participants in all groups, another six ACM participants were excluded from the analyzed cohort. Therefore, a total of 39 participants were included in this study, with 13 participants classified into each of the symptomatic (FAI), asymptomatic (ACM), and control (CTRL) groups (Table 1). This study used a subset of participants who had been enrolled in other studies (Ng et al., 2017; Catelli et al., 2018).

**Table 1:** Summary of demographics, cam morphology measurement of the affected hips, and pain questionnaire, reporting mean ± SD.

**Parameter**		**FAI**	**ACM**	**CTRL**	***P*** **-value**		
Participants (*n*)		13	13	13	–		
Presence of cam morphology (diagnostic imaging)		Yes	Yes	No	–		
Positive impingement test result (clinical signs)		Yes	No	No	–		
Symptoms		Yes	No	No	–		
Age (years)		36 ± 8	34 ± 6	34 ± 7	0.96		
BMI (kg/m^2^)		27 ± 5	26 ± 1	26 ± 3	0.65		
					**FAI vs. ACM**	**FAI vs. CTRL**	**ACM vs. CTRL**
Alpha-angle (deg)	3:00 position	55 ± 6	57 ± 7	43 ± 4	0.66	<0.001	<0.001
	1:30 position	66 ± 4	71 ± 5	53 ± 4	0.01	<0.001	<0.001
HOOS	Symptoms	67 ± 13	96 ± 7	97 ± 6	<0.001	<0.001	0.95
	Pain	67 ± 15	98 ± 5	98 ± 5	<0.001	<0.001	0.98
	Activities of daily living	77 ± 15	100 ± 1	99 ± 2	<0.001	<0.001	0.99
	Sports and recreational activities	55 ± 24	98 ± 7	97 ± 7	<0.001	<0.001	0.97
	Quality of life	38 ± 20	97 ± 9	95 ± 12	<0.001	<0.001	0.95

### Motion Analysis

The pretesting preparation involved completing the hip disability and osteoarthritis outcome score (HOOS) questionnaire (Nilsdotter et al., 2003)—whose scale is 0–100, worst to best outcome score—a 5-min cycle ergometer warm-up and uninstructed lower limb stretching. A total of 45 retroreflective markers were placed on the participants, according to the University of Ottawa motion analysis model marker set (Mantovani and Lamontagne, 2017). Motion capture recorded five deep squatting trials performed at a self-selected pace, with feet positioned parallel at hip-width apart and the arms stretched out anteriorly. One static trial was also recorded per participant, who was asked to assume a T-pose in the middle of the capturing volume, also with their feet parallel at hip-width apart and the palm of their hands facing forward. The marker trajectories were captured using a 10-camera infrared system (200 Hz, MX13, Vicon, UK), and ground reaction forces (GRFs) were captured using two embedded force plates (1,000 Hz, FP4060, Bertec Corporation, OH, USA), with one foot of the participant to each of the force plates. The marker trajectories were labeled and filtered (Woltring, mean squared error = 15 mm^2^) along with the GRF (zero-lag fourth-order low-pass Butterworth filter at 6 Hz) using Nexus 2.6.1 (Vicon, UK). All variables were time normalized with respect to the full squat cycle [from standing to squatted (lowest depth point) and back to standing].

### Musculoskeletal Modeling

The selected musculoskeletal model was customized for the high hip and knee flexion ranges of squatting (Catelli et al., 2019c) and contained 80 lower-limb Hill-type muscle-tendon units (MTUs), with 37 degrees of freedom. All the simulations were conducted in the open-source musculoskeletal simulation software OpenSim 3.3 (Stanford University, Stanford, CA, USA) (Delp et al., 2007). The marker trajectories and GRF data set were converted to OpenSim format (Mantoan et al., 2015), the models were scaled based on static anthropometric dimensions of each patient, recorded during static calibration trial acquisition, and the data were batch processed using a dedicated toolbox (Bedo et al., 2021). Anterior and posterior superior iliac spines, and medial and lateral knee epicondyles markers, were defined according to their placement during the CT scan. Therefore, the markers of the pelvis and knee had their anisotropic scaling weight computed 10 times higher than the other ones of the model. Inverse kinematics and inverse dynamics tools calculated joint angles and internal joint moments for each degree of freedom, while a static optimization tool estimated muscle forces. Once the kinematic state of the model at each time point is known, static optimization resolves the net joint moments into individual muscle forces while minimizing the sum of squared muscle activations. Reserve actuators defined a 10 N optimal force for the three hip coordinates to avoid muscle force saturation during static optimization calculations. The *JointReaction* analysis tool (Steele et al., 2012) calculated HCF as three-dimensional vectors acting on the acetabulum expressed in the femoral coordinate system. The HCF vector direction was also depicted in the sagittal plane. The hip muscle forces and HCF components (x: anterior-posterior, y: superior-inferior, and z: medial-lateral) and their resultant magnitude were normalized to body weight (BW).

### Data Analysis

The muscles that are subdivided in more than one MTU in the model (i.e., adductor magnus, biceps femoris, gluteus maximus, gluteus medius, and gluteus minimus), had their force resultant summed after static optimization, and only the hip muscles have been considered for the analysis.

The demographics and the HOOS questionnaire discrete data were assessed for normality using the Shapiro–Wilk test and the one-way ANOVA followed by *post-hoc* comparisons using Bonferroni corrections (*P* ≤ 0.016).

A one-way ANOVA applied to statistical non-parametric mapping (SnPM) (Pataky, 2010) was used to compare the kinematics, kinetics, muscle forces, and HCF outputs (*P* ≤ 0.016) in the time-normalized full squat cycle (0–100%). The SnPM{t} representing the non-parametric univariate pseudo-t-statistic was calculated at each point of the waveform; if it exceeded the critical threshold *t*, the difference between the groups was considered significant in that part of the waveform. All of the analyses were performed in a custom script (R2018b Matlab, MathWorks, Natick, USA).

## Results

### Demographics and Patient-Reported Outcome Measures

Asymptomatic cam morphology showed HOOS scores comparable with the CTRL, while preoperative patients showed significantly decreased scores in all five categories compared to both the other groups. There were no BMI or age differences among the groups (Table 1).

### Kinematics and Kinetics

**Figure 1:**
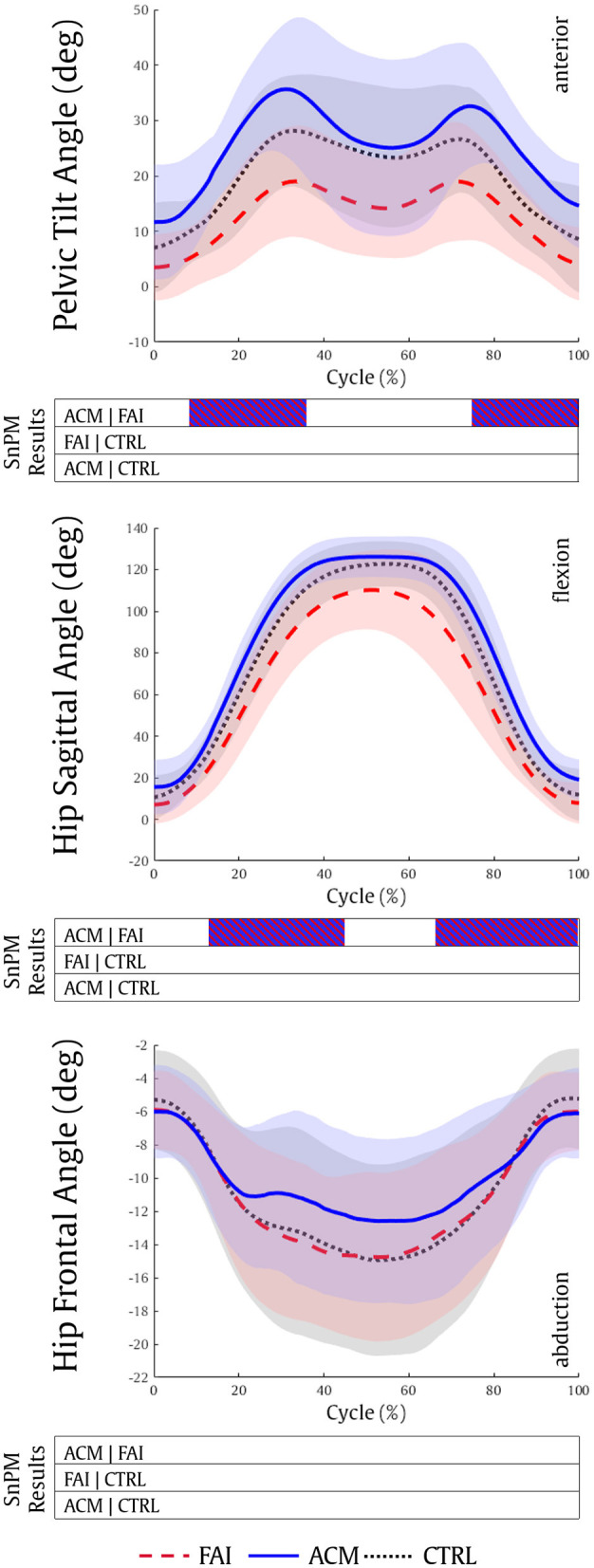
Pelvic tilt, hip sagittal, and hip frontal kinematics during the squatting task for the FAI (dashed red), ACM (solid blue), and CTRL (dotted black) groups. Positive values refer to pelvis anterior, hip flexion and hip adduction kinematics, respectively. SnPM results are displayed below the figure and indicate significant differences among the groups (*P* ≤ 0.016). SnPM, statistical non-parametric mapping; FAI, femoroacetabular impingement; ACM, asymptomatic cam morphology; CTRL, control. Shading indicates standard deviation.

Overall, the ACM has a greater sagittal ROM in the pelvis and the hip (Figure 1). The ACM individuals showed significantly higher anterior pelvic tilt (from 9 to 36%, *P* = 0.001; and 74–100%, *P* = 0.001) and higher hip flexion (13–43%, *P* = 0.001; 66–99%, *P* = 0.002) as compared to patients with symptomatic FAI, during squat. FAI and CTRL comparisons, and ACM and CTRL comparisons, reached no significant differences in pelvis and hip kinematics (*P* > 0.016).

**Figure 2:**
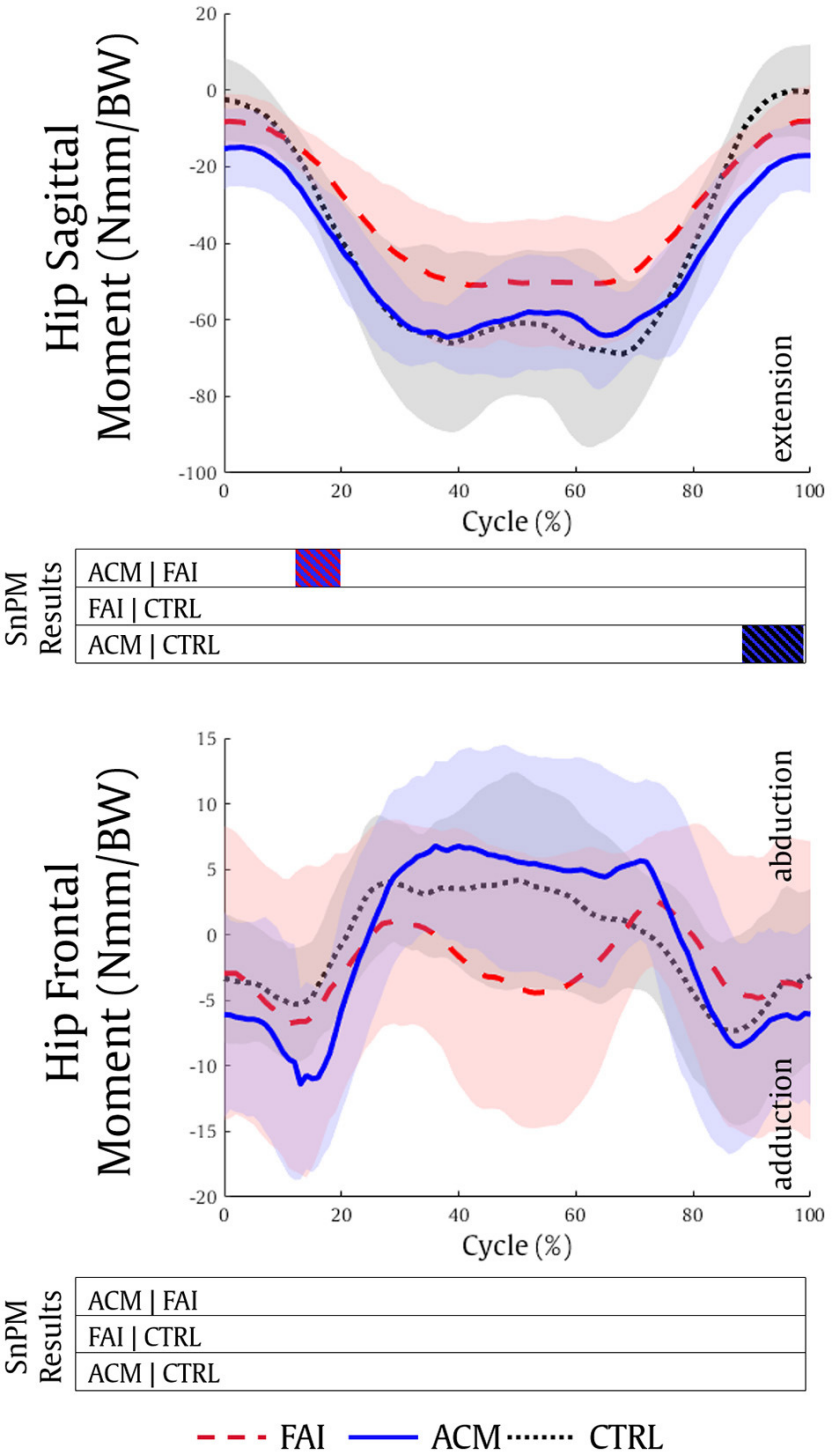
Hip sagittal and hip frontal kinetics during the squatting task for the FAI (dashed red), ACM (solid blue), and CTRL (dotted black) groups. Positive values refer to hip flexion and hip abduction moments, respectively. SnPM results are displayed below the figure and indicate significant differences among the groups (*P* ≤ 0.016). SnPM, statistical non-parametric mapping; BW, body weight; FAI, femoroacetabular impingement; ACM, asymptomatic cam morphology, CTRL, control. Shading indicates standard deviation.

The analysis of the hip kinetics showed a significantly higher hip extension moment in the ACM group compared with both FAI and CTRL groups (12–20%, *P* = 0.001; 88–99%, *P* = 0.001, respectively) (Figure 2). No significant differences in hip kinetics were found between the FAI and CTRL groups (*P* > 0.016), and no significant differences in the hip frontal moment were found among the groups.

### Muscle Forces

The six hip muscle groups that produced more force (>0.25 BW) during mid-squat were selected for plotting: adductor magnus, biceps femoris, gluteus maximus, psoas, rectus femoris, and semimembranosus.

**Figure 3:**
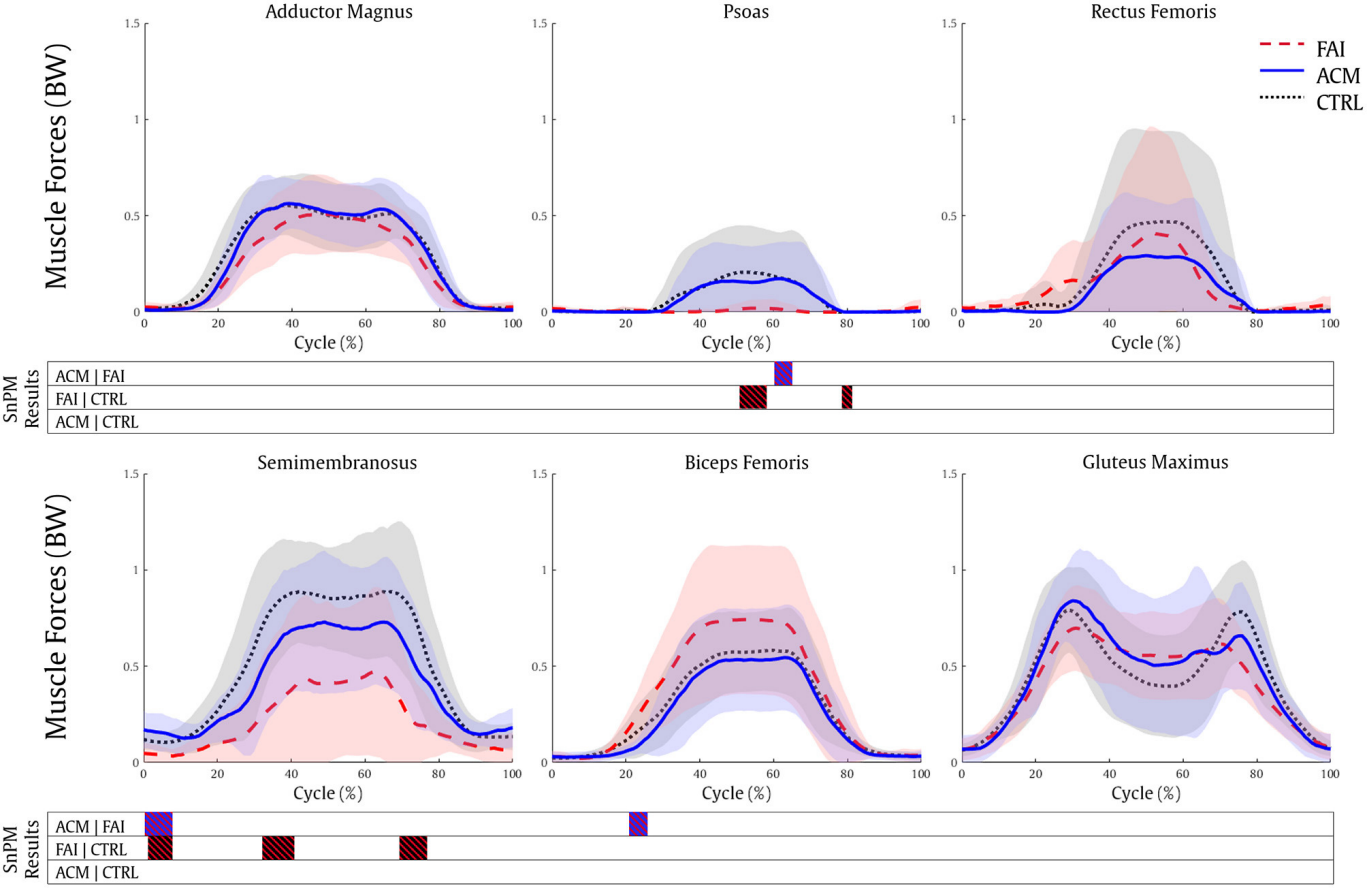
Muscle forces during the squatting task for the FAI (dashed red), ACM (solid blue), and CTRL (dotted black) groups. SnPM results are displayed below the figure and indicate significant differences among the groups (*P* ≤ 0.016). SnPM, statistical non-parametric mapping; BW, body weight; FAI, femoroacetabular impingement; ACM, asymptomatic cam morphology, CTRL, control. Shading indicates standard deviation.

The optimization analyses demonstrated significantly higher psoas (60–64%, *P* = 0.003) and semimembranosus (0–9%, *P* = 0.002) muscle forces in the ACM individuals compared to the FAI individuals. Contrarily, the biceps femoris force was lower in the ACM compared with the FAI (21–26%, *P* = 0.001). The CTRL also showed significantly higher psoas (51–58%, *P* = 0.009 and 79–81%, *P* = 0.002) and semimembranosus (2–9%, *P* = 0.003; 33–41%, *P* = 0.002 and 69–77%, *P* = 0.001) forces compared with the patients with FAI (Figure 3). The ACM showed significantly higher gluteus medius force (16–19%, *P* = 0.006) compared with the CTRL group.

### Hip Contact Forces

**Figure 4:**
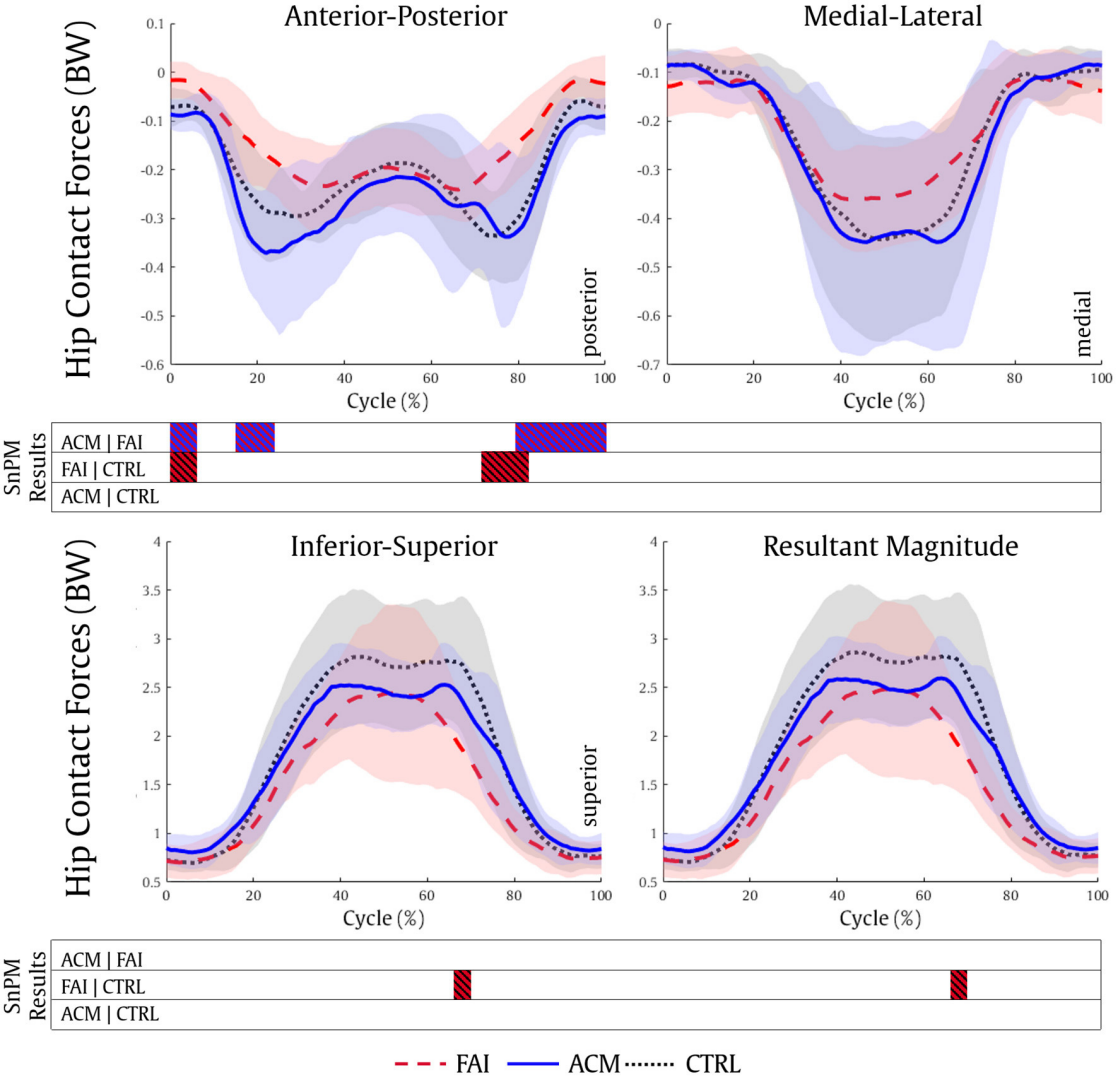
Hip contact forces during the squatting task for the FAI (dashed red), ACM (solid blue), and CTRL (dotted black) groups. SnPM results are displayed below the figure and indicate significant differences among the groups (*P* ≤ 0.016). SnPM, statistical non-parametric mapping; BW, body weight; FAI, femoroacetabular impingement; ACM, asymptomatic cam morphology; CTRL, control. Shading indicates standard deviation.

The ACM individuals showed significantly higher posterior HCF (0–7, 15–26, and 79–100%, all *P* = 0.001) compared with the FAI values. Nonetheless, the CTRL individuals showed higher posterior (0–7%, *P* = 0.005 and 72–82%, *P* = 0.001), superior (67–70%, *P* = 0.005; Figure 4), and total magnitude (67–70%, *P* = 0.006) HCF compared with the FAI. No significant differences in HCF magnitude in any directions were found between the ACM and CTRL groups (*P* > 0.016).

**Figure 5:**
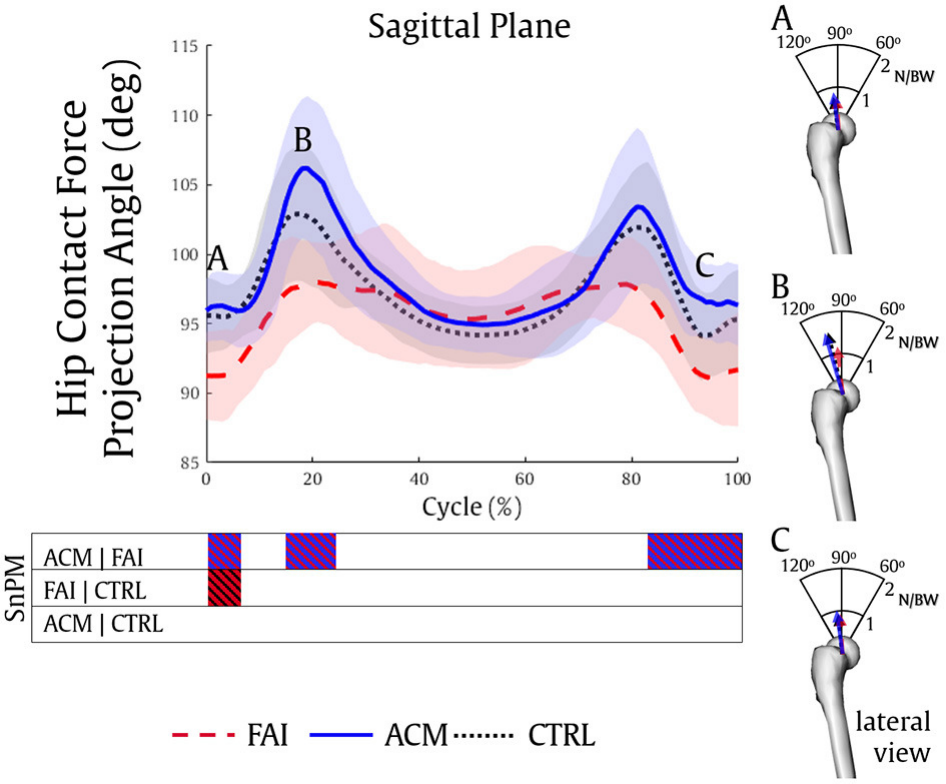
Hip contact forces projection angle during the squatting task in the sagittal plane of a right femur, and three distinct data point scenarios of hip contact forces displayed in the right column **(A–C)** for the FAI (dashed red), ACM (solid blue), and CTRL (dotted black) groups. The FAI demonstrated significantly less posterior forces compared to ACM **(A–C)** and CTRL **(A)**.

Hip contact force vector directions over the sagittal plane were also statistically different between the asymptomatic groups and the FAI. The ACM (0–7, 15–24, and 83–100%, all *P* = 0.001) and the CTRL (0–7%, *P* = 0.002) reached higher contact angles than the FAI during the squat (Figure 5). Once again, no significant differences in HCF vector direction were found between the ACM and the CTRL (*P* > 0.016).

## Discussion

The purpose of this prospective study was to compare the pelvis and hip kinematics, kinetics, muscle forces, and HCF of ACM individuals and compare to both, patients with FAI and CTRL individuals during a deep squat task. The hypotheses were confirmed as the hip kinematics of ACM individuals resembled the CTRL and reached higher angles of anterior pelvic tilt and hip flexion at descent and ascent mid-phases of the squat cycle compared with the patients with FAI. The ACM individuals also showed greater hip extension moments during the descent and ascent phases compared with the FAI and CTRL, respectively. While muscle force estimations did not vary between the ACM and the CTRL individuals, they significantly differed from the FAI values. Different from our hypotheses, the HCF analysis showed similar contact loads between the ACM and the CTRL, which were significantly greater compared with the patients with FAI. The findings in the ACM individuals go in line with a previous study (Catelli et al., 2020), showing that the HCF reached a load magnitude of 2.5–3 times BW at the deepest phase of the squat, and the adductor magnus, quadriceps, hamstrings, and glutei muscles being the main contributors to perform such a task. To our knowledge, this is the first study that compared hip muscle and contact forces estimations in ACM with symptomatic FAI and healthy participants during a deep squatting task using musculoskeletal modeling. These results show evidence that despite the cam-type morphology, ACM individuals relied on a biomechanical strategy that resembled the CTRL individuals, and are unrelated to the muscle force imbalance that seems to be present in patients with symptomatic FAI (Lamontagne et al., 2015).

Although the presence of a cam-type hip morphology increases the risk of labral tears and progressive hip joint degeneration (Agricola et al., 2013), differences in gait biomechanics between FAI and CTRL are not well-explained by the size of cam morphology alone (Savage et al., 2021). ACM individuals demonstrated higher anterior pelvic tilt compared to their symptomatic peers, which aligned well with previous reports that have also highlighted stronger hip extensors in the asymptomatic individuals (Catelli et al., 2018; Ng et al., 2018a). These two factors can indeed provide the ACM with a “closer to normal” pelvis kinematics during the squat cycle. When considering that the cam-type morphology is located at the anterior-superior portion of the femoral head (Chakraverty et al., 2013), it would be inclined to impinge when there is hip flexion combined with an anteriorly tilted pelvis (Ross et al., 2014). The higher degrees of both hip flexion and anterior pelvic tilt reached by the ACM individuals may provide additional evidence that it is not only the presence or size of the cam morphology that modifies joint biomechanics in patients with FAI but it might be related to a protective sensory-motor strategy to avoid pain (Lamontagne et al., 2015; Diamond et al., 2017, 2019; Savage et al., 2021). The progression of the cam-type morphology associated with the beginning of the symptoms could have induced patients with FAI to adopt a more posteriorly tilted pelvis position, which would limit the hip mobility, therefore establishing a protective mechanism to reduce HCFs (Ng et al., 2018b; Catelli et al., 2019b, 2020, 2021).

Anatomical parameters have also been associated with the FAI syndrome symptomatology. Decreased femoral neck-shaft angle associated with reduced pelvic mobility (Ng et al., 2015), greater pelvic incidence, and greater acetabular version (Grammatopoulos et al., 2018) can discriminate those symptomatic FAI to an asymptomatic ACM cohort. Nonetheless, a higher pelvic incidence can also serve as a predictor for limited sagittal hip mobility (Ng et al., 2018a). Therefore, the presence of the cam morphology combined with other anatomical parameters cannot completely explain clinical symptoms or decreased hip and pelvis ROM (Ng et al., 2018a), once again suggesting that altered muscle functions may play an important role in the FAI symptomatology. The similar hip flexion moment pattern produced by ACM and CTRL (Figure 2) is indicative of the muscle force production similarities between these individuals compared with the FAI. This can be explained by the FAI reduced muscle force of one of the main hip flexors: the psoas. Its estimated force magnitude was significantly lower in the patients with FAI, while it ranged ~0.2 BW in the ACM and CTRL (Figure 3), can be associated with decreased anterior pelvis tilt and hip flexion in the symptomatic patients (Ng et al., 2018a,b; Catelli et al., 2019b). The direct role of the iliopsoas on the FAI pathomechanism is yet to be established, but studies (Ng et al., 2018b) suggested that a tighter iliopsoas tendon produces unfavorable stresses to the anterior capsulolabral complex during hip extension (Lewis et al., 2007; Alpert et al., 2009; Kennedy et al., 2009), likely leading to labral damage (Domb et al., 2011).

The semimembranosus was the other muscle that also has demonstrated significantly lower forces in the FAI compared to both ACM and CTRL. Acting synergistically with the gluteus maximus to support posterior pelvic tilt, a decreased semimembranosus force could be associated with a protective sensory-motor strategy during squatting (Catelli et al., 2020). In fact, the higher biceps femoris forces produced by the FAI (compared to ACM, 21–26%) suggest that the symptomatic patients favor loading the biceps femoris in contrast with its medial neighbor, the semimembranosus. This could also suggest that these individuals would reach a higher hip abduction, which did not happen in our analysis, perhaps due to the lower hip abduction strength seen in FAI (Casartelli et al., 2011). At the mid-phase of the squat, during its lowest depth, there is a posterior tilt of the pelvis that is reached by high assistance of the hamstrings. The imbalanced hamstrings in the patients with FAI are suggestive of a muscular adaptive mechanism to avoid the pain that affects the pelvis kinematics during the squat. Additionally, although the gluteus maximus did not present significant differences among our groups, a higher force production of its inferior portion during a squat has already been reported (Catelli et al., 2020), and along with the decreased semimembranosus forces, is also suggestive of imbalance in hip muscle force production. Stronger hip extensors play an important role in the sagittal pelvic ROM, allowing the ACM individuals to posteriorly tilt their pelvis while reaching the bottom of the squat. Rehabilitation plans that improve hip extensors strengthening and enhancement of pelvis mobility may alleviate symptoms for patients with FAI (Catelli et al., 2018).

Compared with CTRL individuals, lower magnitude HCF in those with symptomatic FAI has already been reported during gait (Ng et al., 2018b; Catelli et al., 2019b) and squat (Catelli et al., 2020). Our results showed that the ACM individuals also show significantly higher posterior HCF during both phases of the squat (descent and ascent) compared with their symptomatic peers, and resembling CTRL outputs. Considering the muscle forces play an important role in contributing to HCF estimations (Correa et al., 2010), the stronger ACM muscles (Catelli et al., 2018) assisted these individuals to reach higher HCF levels (Figure 4). Concurrently, the differences in muscle forces and HCF vector direction can be attributed to the different joint positioning (hip and pelvis) during the squat among the groups (Figures 4, 5), especially when considering the greater anterior pelvis tilt reached by the ACM during the task.

The use of a maximal squat test as a diagnostic tool for assessing FAI in the clinical setting has already been proposed (Ayeni et al., 2014). The idea of using a task that demands large hip and pelvis mobility that discriminates individuals with the cam-type morphology from their cam-free peers is understandable as it has already been shown that patients with FAI are unable to squat as deep as their healthy peers because of mobility restrictions at the pelvis and hip (Lamontagne et al., 2009; Catelli et al., 2018). However, when including asymptotic individuals in the analysis, the ACM kinematics resembles the CTRL group, with higher hip flexion and anterior pelvic tilt, refuting the concept that the restriction in mobility was only caused by the presence of femoral cam morphology. This reinforces the speculation that muscle contraction imbalance (Catelli et al., 2018) combined with hip instability due to weak capsule (Ng et al., 2021) could be associated with FAI symptomatology.

The limitations to this study include the small sample size of our cohort, as increasing the number of participants would have resulted in higher predictive power. Limiting our cohort to male individuals with cam-type morphology only, limits our findings to this population, as differences by sex may occur (Lewis et al., 2018; Brown-Taylor et al., 2020). Second, static optimization can underestimate muscle force production during co-contractions that are modified by a joint pathology; however, this technique still produces results closest to experimental HCF (Wesseling et al., 2015). Third, the used models did not include subject-specific geometries of the cam morphology, once each participant had his musculoskeletal data scaled based on a cam-free generic model. A model that presents subject-specific cam morphology may produce different HCF outputs.

This study provided insights into muscle forces and HCF in the ACM male population, showing that both muscle forces and HCFs with the greater pelvis and hip kinematics are closely comparable with the controls, rather than their FAI syndrome peers. These results rebut the concept that FAI symptomatology is caused by the presence of the cam morphology only but also associated with different muscle contraction strategies inducing hip instability. Future studies are necessary to better understand the complex role of soft tissues, muscle contraction pattern, and divergence of hip anatomical parameters.

## Data Availability Statement

The raw data supporting the conclusions of this article are currently being stored at the University of Ottawa, Canada. It is available upon request.

## Ethics Statement

The studies involving human participants were reviewed and approved by The Ottawa Hospital and the University of Ottawa Research Ethics Boards. The patients/participants provided their written informed consent to participate in this study.

## Author Contributions

DC, PB, and ML conceptualized the study. DC and EK processed and analyzed the data. DC, EK, and ML interpreted the data, and also drafted and revised the manuscript. All authors read and approved the final manuscript.

## Conflict of Interest

The authors declare that the research was conducted in the absence of any commercial or financial relationships that could be construed as a potential conflict of interest.

## Publisher's Note

All claims expressed in this article are solely those of the authors and do not necessarily represent those of their affiliated organizations, or those of the publisher, the editors and the reviewers. Any product that may be evaluated in this article, or claim that may be made by its manufacturer, is not guaranteed or endorsed by the publisher.
